# Potential of newly isolated strain *Pseudomonas aeruginosa* MC-1/23 for the bioremediation of soil contaminated with selected non-steroidal anti-inflammatory drugs

**DOI:** 10.3389/fmicb.2025.1542875

**Published:** 2025-03-03

**Authors:** Magdalena Klim, Agnieszka Żmijowska, Mariusz Cycoń

**Affiliations:** ^1^Department of Microbiology, Faculty of Pharmaceutical Sciences, Medical University of Silesia, Sosnowiec, Poland; ^2^Ecotoxicology Research Group, Laboratory of Analytical Chemistry, Łukasiewicz Research Network–Institute of Industrial Organic Chemistry Branch Pszczyna, Warsaw, Poland

**Keywords:** *Pseudomonas aeruginosa*, NSAIDs, ibuprofen, diclofenac, naproxen, bioaugmentation, soil bioremediation

## Abstract

The widespread usage of non-steroidal anti-inflammatory drugs (NSAIDs) has resulted in their significant accumulation in the environment, necessitating the development of effective methods for their removal. This study primarily isolated a bacterial strain capable of degrading specific NSAIDs and evaluated its potential for eliminating these drugs from contaminated soil through bioaugmentation. The objectives were achieved by assessing the degradation rates of ibuprofen (IBF), diclofenac (DCF), and naproxen (NPX) in liquid media and soil samples inoculated with a newly identified strain, *Pseudomonas aeruginosa* MC-1/23. In addition, the effect of natural soil microflora and abiotic conditions on the breakdown of the tested NSAIDs was examined. The findings revealed that strain MC-1/23 could metabolize these compounds in a mineral salt medium, utilizing them as carbon and energy sources, suggesting metabolic degradation. When nonsterile soil was augmented with the *P. aeruginosa* MC-1/23 strain, the degradation rates of the drugs significantly improved, as evidenced by reductions in *t*_1/2_ values by 5.3-, 1.4-, and 5.8-fold for IBF, DCF, and NPX, respectively, compared with soil containing only natural microflora. These results confirm that the introduced strain enhances the catabolic potential of existing microflora. Thus, the strain’s degradation and bioremediation capabilities offer valuable applications for remediating NSAID-contaminated soils.

## 1 Introduction

A vast array of pharmaceuticals is utilized globally each year in both human and veterinary medicine, with non-steroidal anti-inflammatory drugs (NSAIDs) being among the most commonly used. Notable examples of widely consumed NSAIDs include ibuprofen (IBF), diclofenac (DCF), and naproxen (NPX). The widespread availability of NSAIDs as over-the-counter medications makes it challenging to accurately quantify their consumption levels (Tran et al., 2018; Dolu and Nas, 2023). Furthermore, their use as supplementary treatments during the COVID-19 pandemic substantially increased global NSAID usage (Wojcieszyńska et al., 2022), consequently elevating their environmental concentrations (Parida et al., 2022; Zhao et al., 2024).

In living organisms, NSAIDs are not fully degraded but undergo minimal transformation, leading to their excretion in nearly unchanged forms. Once released, these compounds enter sewage systems and eventually wastewater treatment plants (WWTPs). Research indicates that WWTPs are incapable of completely eliminating NSAIDs from wastewater, resulting in their accumulation in sewage sludge. This sludge, often repurposed as fertilizer, introduces NSAIDs into surface water, soil, and even drinking water, where they can re-enter living organisms and accumulate (Márta et al., 2018; Dolu and Nas, 2023; Bean et al., 2024; Zhao et al., 2024). Additionally, improper storage and disposal of expired or unused NSAIDs contribute to environmental contamination. These substances may directly enter WWTPs or landfills and subsequently contaminate groundwater and soil (Stackelberg et al., 2004; Williams and Mclain, 2012; Boxall and Brooks, 2024). Such processes elevate NSAID concentrations in the environment, posing significant risks to the ecological balance of various ecosystems (Zorita et al., 2009; Cycoń et al., 2016).

Currently, no specific parameters or regulatory limits have been established for NSAIDs in the soil environment. Studies have shown that NSAID concentrations in soil vary significantly across different countries, influenced by the type of environmental sample analyzed. NSAIDs have been detected in soils at varying concentrations, highlighting the influence of factors such as sludge application frequency, soil characteristics, precipitation, and runoff (Verlicchi and Zambello, 2015; Sadutto et al., 2021). For example, Aznar et al. (2014) reported soil concentrations of IBF and NPX at 15 and 59 μg/kg, respectively, whereas other NSAIDs, including ketoprofen (KTP) and DCF, were undetected in the same samples. Conversely, a separate study identified substantially higher concentrations in soil, reporting levels of 610, 257, and 199 μg/kg for IBF, DCF, and NPX, respectively (Ashfaq et al., 2017).

Variations in the water solubility and mobility of pharmaceutical compounds, coupled with soil properties, influence the behavior of NSAIDs in the soil. These compounds may adhere to soil particles, seep into groundwater (Topp et al., 2008; Kidd et al., 2024), or be transported to surface water, undergoing diverse physical, chemical, or biochemical transformations (Beausse, 2004; Monteiro and Boxall, 2010; Dolu and Nas, 2023; Lin et al., 2023). Among the key mechanisms driving NSAID dissipation in soil are microbial processes, with the natural microflora exhibiting significant potential for drug degradation (Xu et al., 2009; Girardi et al., 2013). However, the efficiency of these degradation processes is heavily influenced by environmental factors such as soil moisture, temperature, and type (Topp et al., 2008; Carr et al., 2011; Shu et al., 2021; Lin et al., 2023; Shu et al., 2023).

Biodegradation and environmental self-cleaning, primarily driven by microorganisms, are critical for mitigating contamination (Singer et al., 2005; Topp et al., 2008; Cycoń et al., 2017; Muter, 2023). Research efforts are increasingly directed toward harnessing bacterial and fungal strains for bioremediation of environments polluted with various chemical compounds. Studies have demonstrated that microorganisms can degrade NSAIDs through metabolic pathways, where the compounds serve as carbon and energy sources, or co-metabolic pathways, which rely on an external, readily degradable organic compound. Metabolic and co-metabolic degradation capabilities have been documented in fungal genera such as *Phanerochaete* (Hata et al., 2010; Rodarte-Morales et al., 2012; Yin et al., 2024), *Trametes versicolor* (Marco-Urrea et al., 2010; Rodríguez-Rodríguez et al., 2010; Tormo-Budowski et al., 2021), and *Cunninghamella* (Zhong et al., 2003), as well as bacterial genera including *Sphingomonas* (Murdoch and Hay, 2005), *Bacillus* (Marchlewicz et al., 2016), *Pseudomonas* (Ahmed et al., 2001; De Gusseme et al., 2011; Poddar et al., 2024), *Nocardia* (Chen and Rosazza, 1994), *Delftia* (De Gusseme et al., 2011), *Rhodococcus* (Ivshina et al., 2006), *Patulibacter* (Almeida et al., 2013), Var*iovorax* (Murdoch and Hay, 2015), *Planococcus* (Domaradzka et al., 2015; Dzionek et al., 2024), and *Stenotrophomonas* (Zhang et al., 2013; Wojcieszyńska et al., 2014). However, these findings are largely confined to laboratory studies using liquid media, and there is a notable lack of reports on the application of microorganisms for bioremediating NSAID-contaminated soils. Accordingly, this study aimed to isolate a bacterial strain capable of degrading specific NSAIDs and evaluate its efficacy in removing these compounds from contaminated soil via bioaugmentation. The objectives were addressed by measuring the degradation rates of IBF, DCF, and NPX in liquid media and soil samples inoculated with the bacterial strain. Additionally, the role of natural soil microflora and abiotic conditions in the degradation of NSAIDs was investigated.

## 2 Materials and methods

### 2.1 Chemicals

The experiment utilized three European Pharmacopeia reference standards of NSAIDs sourced from Merck (Germany), along with commercially available NSAIDs. The reference standards included IBF, DCF as sodium salt, and NPX. The commercially available formulations were Ibuprom (containing 200 mg of IBF and additional ingredients), Diclac Duo 150 (comprising 150 mg of DCF sodium salt and additional components), and NPX 500 (with 500 mg of NPX and additional ingredients). The fundamental physicochemical properties of the NSAIDs used are summarized in Table 1, whereas the compositions of the commercial formulations, as provided by the manufacturers, are detailed in Supplementary Table S1. All additional chemicals and solvents used in the study were of high-performance liquid chromatography grade and also procured from Merck, Germany.

**Table 1:** Basic physicochemical properties of NSAIDs used in the experiment.

Parameter	Ibuprofen	Diclofenac sodium	Naproxen
Chemical formula	C_13_H_18_O_2_	C_14_H_10_Cl_2_NNaO_2_	C_14_H_14_O_3_
IUPAC name	2-[4-(2-methylpropyl) phenyl]propanoic acid	sodium;2-[2-(2,6-dichloroanilino) phenyl]acetate	(2S)-2-(6-methoxynaphthalen-2-yl)propanoic acid
Chemical structure	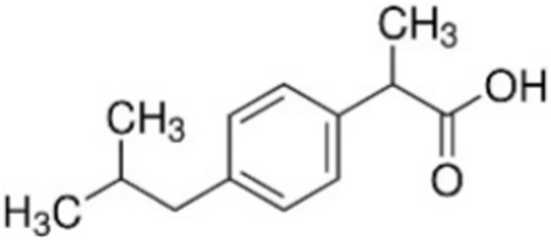	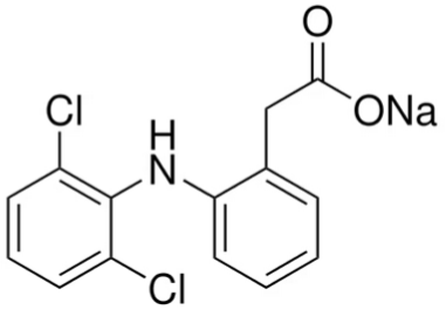	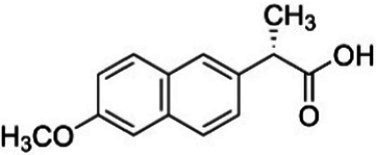
Molecular weight (g/mol)	206.28	296.15	230.26
Water solubility	21 mg/L	14 mg/mL	15.9 mg/L
Log *K*_OW_	3.97	4.51	3.18
pK_a_	4.45	3.99	4.18

### 2.2 Isolation procedure of bacterial strain

**Figure 1:**
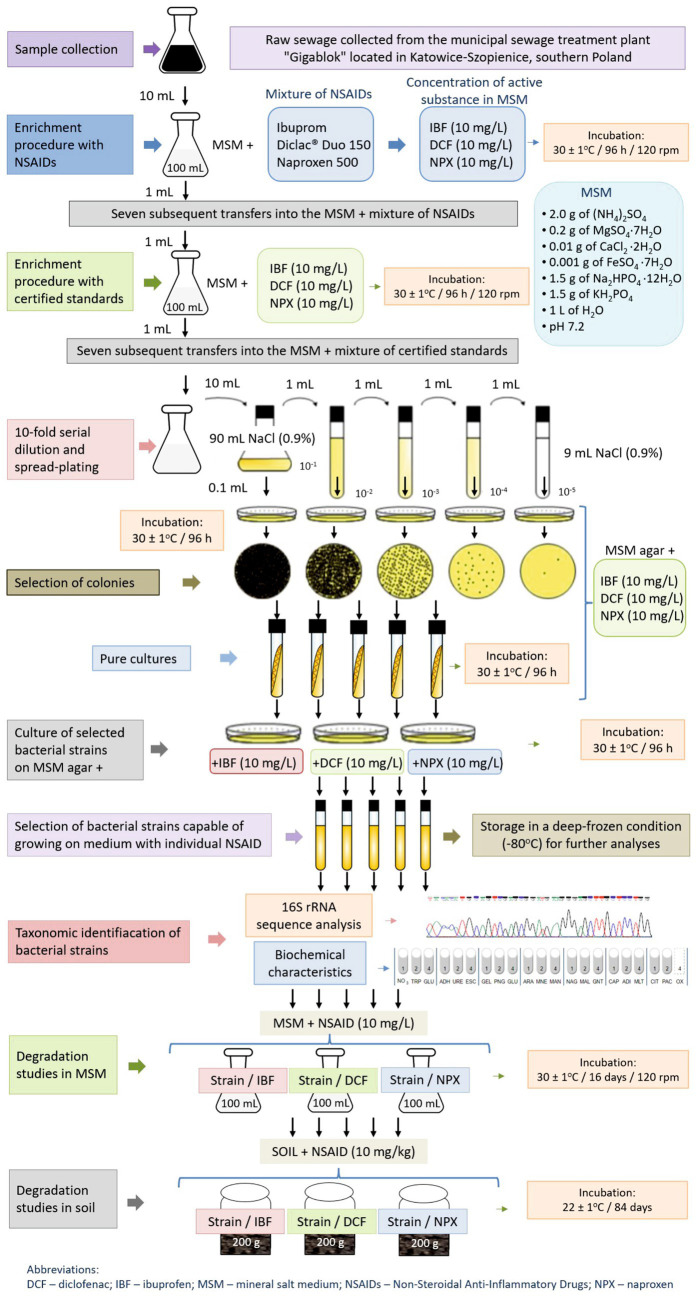
Test procedures used in the experiment including isolation and identification of bacterial strain and degradation studies of selected NSAIDs in liquid medium and soil.

The bacterial strain capable of degrading the selected drugs was isolated from raw sewage obtained from the municipal sewage treatment plant “Gigablok” in Katowice-Szopienice, southern Poland. The isolation process employed a two-step enrichment procedure using a mineral salt medium (MSM), the composition of which is shown in Supplementary Table S2. The initial stage of isolation involved enriched cultivation in MSM supplemented with a mixture of commercially available NSAIDs. In the second phase of isolation, the procedure was conducted using MSM and then MSM agar supplemented with reference standards of IBF, DCF, and NPX at a concentration of 10 mg/L each. The use of relatively high concentrations of drugs during the screening procedure was aimed to isolate a bacterial strain with a really high potential to degrade the tested NSAIDs, and whose growth would not be limited by high doses of the drugs tested. The obtained distinct colonies on MSM agar were purified by repeated streaking on the same medium. Ultimately, a strain capable of growing on MSM agar containing each of the tested drugs was successfully selected. The procedure of isolation of bacterial strain is presented in Figure 1 and detailed in the Supplementary materials.

### 2.3 Identification of bacterial strain

Characterization and identification of the isolated strain were performed through biochemical testing and 16S rRNA gene analysis (Figure 1). The biochemical properties and substrate utilization profile of the isolate were assessed using the API 20 NE System (bioMérieux Inc., France) following the manufacturer’s instructions. For 16S rRNA sequence analysis, genomic DNA was extracted from a culture harvested during the late exponential growth phase using the GeneMATRIX Bacterial and Yeast Genomic DNA Purification Kit (Eurx, Poland) as per the manufacturer’s protocol. The 16S rRNA gene was amplified using a PCR mix containing the universal primer pair: 27f and 1492r (Lane, 1991) and other components (Supplementary Table S3). Amplification was carried out with a PCR Master Mix Kit (Promega) in a PTC-118 Thermal Cycler (Bio-Rad, CA, USA) under specified conditions (Supplementary Table S4). The amplified products were purified using the GeneMATRIX PCR/DNA Clean-Up Purification Kit (Eurx, Poland) before sequencing. Gene sequencing was performed using a Big Dye Terminator Cycle Sequencing Kit (Applied Biosystem) on an AbiPrism^®^3,100 Genetic Analyzer. The resulting sequences were compared with known 16S rRNA gene sequences via the BLAST server hosted by the National Center for Biotechnology Information. Sequence alignment was conducted using CLUSTAL W, and phylogenetic analysis was performed using the neighbor-joining (NJ) method in MEGA version 11 software. The characterized and identified bacterial strain, capable of growing on MSM agar with each of the tested drugs, was employed in further experiments investigating drug degradation in liquid media and soil.

### 2.4 Study of the degradation of NSAIDs

#### 2.4.1 Study of the degradation of NSAIDs in MSM

**Figure 2:**
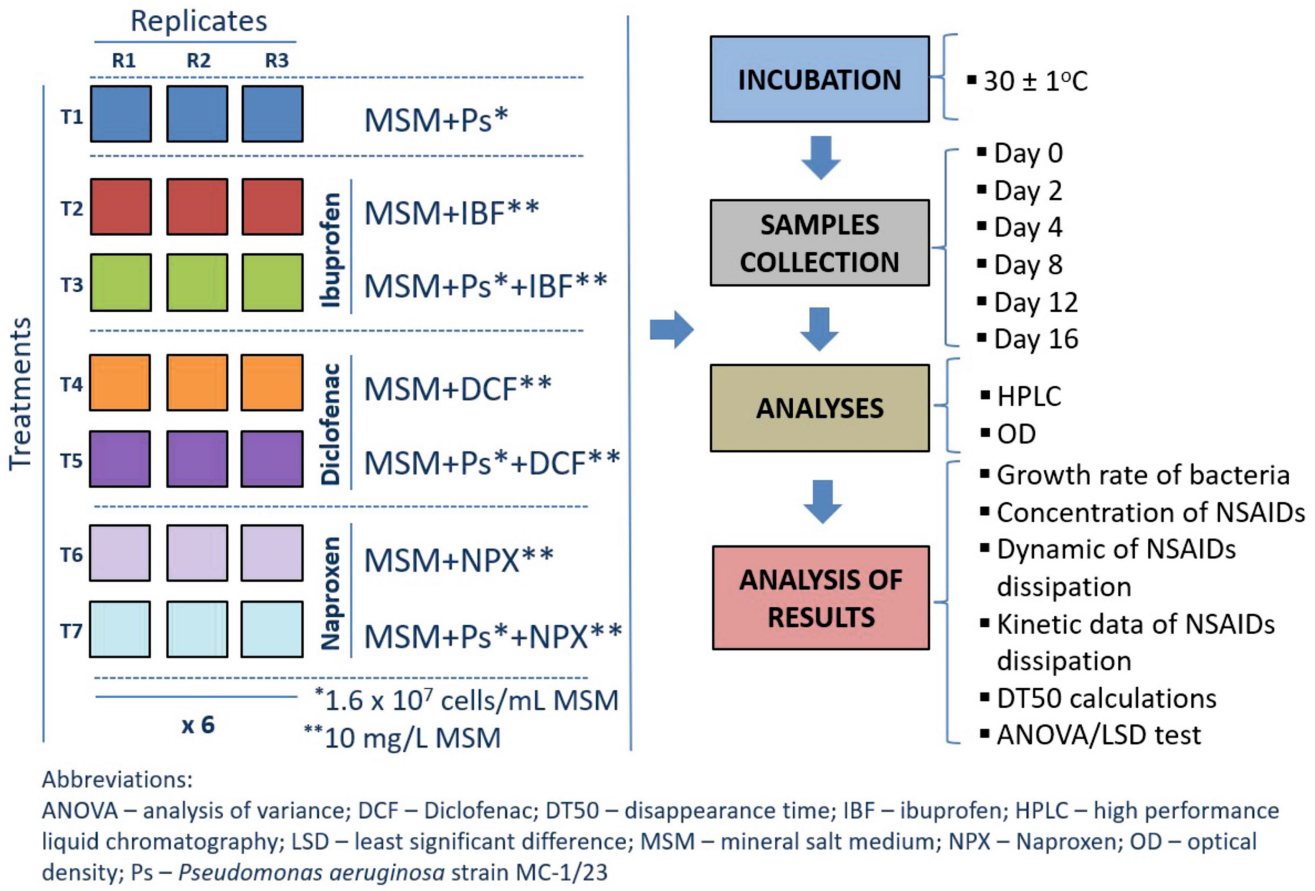
Design and performed analyses for the experiment with mineral salt medium.

Degradation experiments were conducted in 200-mL Erlenmeyer flasks, each containing 100 mL of sterile MSM supplemented with individual reference standards of NSAIDs as the sole carbon source. The flasks were inoculated with the bacterial strain (*Pseudomonas aeruginosa* MC-1/23) under the conditions MSM + Ps + IBF, MSM + Ps + DCF, and MSM + Ps + NPX. Control samples included MSM + Ps and MSM with individual NSAIDs (IBF, DCF, or NPX). The drugs were added to achieve a final concentration of 10 mg/L in the medium. To prepare the inoculated samples, a bacterial suspension was introduced to the MSM to reach a final bacterial count of approximately 1.6 × 10^7^ cells/L. A detailed description of the inoculum preparation is available in the Supplementary materials. The experiment was designed as a completely randomized block with three replications for each treatment across six sampling times, resulting in a total of 126 flasks (seven treatments × three replications × six sampling times). The experimental design and analytical procedures for the MSM-based study are illustrated in Figure 2. The experiment had the following treatments:

- MSM + Ps – mineral salt medium with *Pseudomonas aeruginosa* strain MC-1/23 (1.6 × 10^7^ cells/mL): 18 flask × 100 mL; for analysis – 3 replicates × 6 sampling times,
- MSM + IBF – mineral salt medium with ibuprofen (10 mg/L): 18 flask × 100 mL; for analysis – 3 replicates × 6 sampling times,
- MSM + Ps + IBF – mineral salt medium with *Pseudomonas aeruginosa* strain MC-1/23 (1.6 × 10^7^ cells/mL) and ibuprofen (10 mg/L): 18 flask × 100 mL; for analysis – 3 replicates × 6 sampling times,
- MSM + DCF – mineral salt medium with diclofenac (10 mg/L): 18 flask × 100 mL; for analysis – 3 replicates × 6 sampling times,
- MSM + Ps + DCF – mineral salt medium with *Pseudomonas aeruginosa* strain MC-1/23 (1.6 × 10^7^ cells/mL) and diclofenac (10 mg/L): 18 flask × 100 mL; for analysis – 3 replicates × 6 sampling times,
- MSM + NPX – mineral salt medium with naproxen (10 mg/L): 18 flask × 100 mL; for analysis – 3 replicates × 6 sampling times,
- MSM + Ps + NPX – mineral salt medium with *Pseudomonas aeruginosa* strain MC-1/23 (1.6 × 10^7^ cells/mL) and naproxen (10 mg/L): 18 flask × 100 mL; for analysis – 3 replicates × 6 sampling times.

The flasks were incubated on a rotary shaker set to 120 rpm in a darkened thermostatic chamber maintained at 30 ± 1°C. At random intervals, samples were withdrawn to assess bacterial growth rates and to perform chemical analyses for determining the concentrations of IBF, DCF, and NPX. The growth of the bacterial strain was monitored spectrophotometrically by measuring the optical density (OD) at 660 nm using a UV–VIS spectrophotometer (Varian, USA).

#### 2.4.2 Study of the degradation of NSAIDs in soil

**Figure 3:**
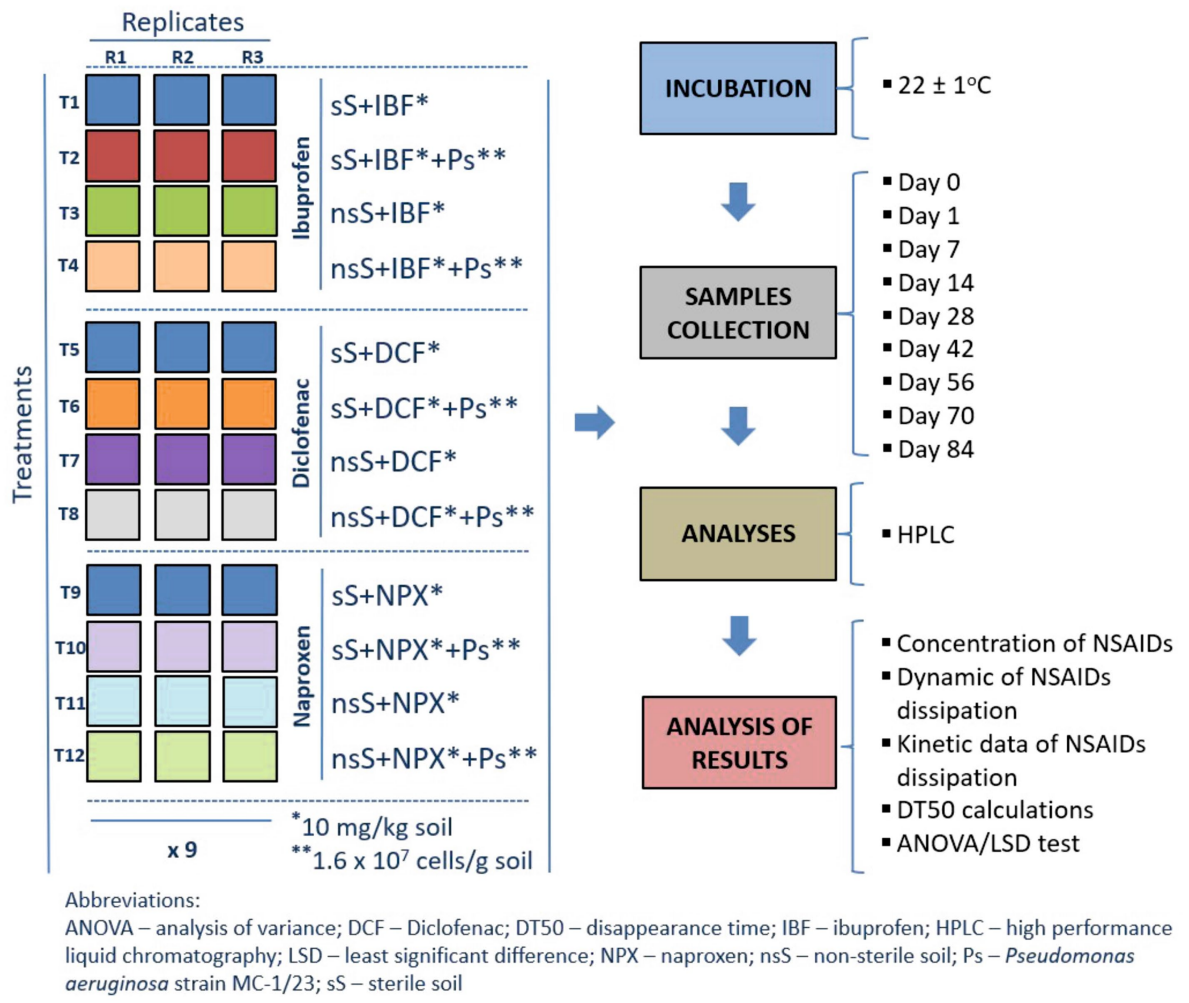
Design and performed analyses for the experiment with soil.

Soil samples were collected from the top 0–20 cm layer of grass-covered private fields near Żywiec, southern Poland. The soil used in the study was not subjected to any agrotechnical treatment with either organic or inorganic chemicals for the last 10 years prior to sampling. Also, no anthropogenic contamination was documented during this period. In the laboratory, the soil was sieved to a particle size of <2 mm and used immediately for the experiment. The physicochemical properties of the soil were determined according to ISO standards, as detailed in Table 2, and the soil was classified as loamy sand based on the US/FAO system (Cycoń et al., 2010; Orlewska et al., 2018). The rate of NSAID degradation was evaluated under abiotic and biotic conditions using sterile and nonsterile soils, respectively. Sterilization was performed three times, each for 1 h at 121°C. The experiment followed a completely randomized block design with three replicates per treatment across nine sampling times, resulting in four treatments × three replicates × nine sampling times for each NSAID. The experimental design and analytical procedures for the soil-based study are illustrated in Figure 3. The experiment had the following treatments:

**Table 2:** Characteristics of the soil used in the experiment.

Parameter	Value	Method of determination	References
Sand (2000–50 μm) (%)	70.0 ± 2.2	Sedimentation and sieving method	ISO 11277 (2020)
Silt (<50–2 μm) (%)	22.0 ± 1.3		
Clay (<2 μm) (%)	8.0 ± 1.1		
Density g/cm^3^	1.23 ± 0.07		
pH(in water) (1:5)	6.8 ± 0.2	Measurement with glass electrode	ISO 10390 (2021)
Cation exchange capacity (CEC) (cmol + /kg)	11.0 ± 0.7	Modified Gillman method	ISO 11260 (2018)
Water holding capacity (WHC) (%)	38.0 ± 1.3	Gravimetric method	ISO 14239 (2017)
C_org_ (%)	1.2 ± 0.1	Oxidation in the presence of H_2_SO_4_	ISO 23400 (2021)
N_tot_ (%)	0.13 ± 0.02	Modified Kjeldahl method	ISO 11261 (1995)
Microbial biomass (mg/kg)	854 ± 16	Substrate-induced respiration (SIR)	ISO 14240-1 (1997)
Soil texture classification	loamy sand	–	US/FAO System

The values are the means of three replicates ± standard deviation, which was within 5% of the mean.

- sS + IBF – sterile soil with ibuprofen (10 mg/kg): 5,400 g (27 pots × 200 g); for analysis – 3 replicates × 9 sampling times,
- sS + IBF + Ps – sterile soil with ibuprofen (10 mg/kg) and *Pseudomonas aeruginosa* strain MC-1/23 (1.6 × 10^7^ cells/ g): 5,400 g (27 pots × 200 g); for analysis – 3 replicates × 9 sampling times,
- nsS + IBF – non-sterile soil with ibuprofen (10 mg/kg): 5,400 g (27 pots × 200 g); for analysis – 3 replicates × 9 sampling times,
- nsS + IBF + Ps – non-sterile soil with ibuprofen (10 mg/kg) and *Pseudomonas aeruginosa* strain MC-1/23 (1.6 × 10^7^ cells/ g): 5,400 g (27 pots × 200 g); for analysis – 3 replicates × 9 sampling times,
- sS + DCF – sterile soil with diclofenac (10 mg/kg): 5,400 g (27 pots × 200 g); for analysis – 3 replicates × 9 sampling times,
- sS + DCF + Ps – sterile soil with diclofenac (10 mg/kg) and *Pseudomonas aeruginosa* strain MC-1/23 (1.6 × 10^7^ cells/ g): 5,400 g (27 pots × 200 g); for analysis – 3 replicates × 9 sampling times,
- nsS + DCF – non-sterile soil with diclofenac (10 mg/kg): 5,400 g (27 pots × 200 g); for analysis – 3 replicates × 9 sampling times,
- nsS + DCF + Ps – non-sterile soil with diclofenac (10 mg/kg) and *Pseudomonas aeruginosa* strain MC-1/23 (1.6 × 10^7^ cells/g): 5,400 g (27 pots × 200 g); for analysis – 3 replicates × 9 sampling times,
- sS + NPX – sterile soil with naproxen (10 mg/kg): 5,400 g (27 pots × 200 g); for analysis – 3 replicates × 9 sampling times,
- sS + NPX + Ps – sterile soil with naproxen (10 mg/kg) and *Pseudomonas aeruginosa* strain MC-1/23 (1.6 × 10^7^ cells/ g): 5,400 g (27 pots × 200 g); for analysis – 3 replicates × 9 sampling times,
- nsS + NPX – non-sterile soil with naproxen (10 mg/kg): 5,400 g (27 pots × 200 g); for analysis – 3 replicates × 9 sampling times,
- nsS + NPX + Ps – non-sterile soil with naproxen (10 mg/kg) and *Pseudomonas aeruginosa* strain MC-1/23 (1.6 × 10^7^ cells/g): 5,400 g (27 pots × 200 g); for analysis – 3 replicates × 9 sampling times.
- The individual NSAID reference standards as powder were mixed with sterile quartz sand (<0.5 mm) and thoroughly incorporated into the soil (50 g/kg) to achieve a final concentration of 10 mg/kg soil. The use of a high initial concentration of the NSAIDs tested in the experiment with soil reflected the most adverse scenarios associated with the entry of large quantities of drugs into the soil as a result of the uncontrolled disposal of unused drugs into municipal waste or depositing them in landfills, and was also intended to evaluate the potential hazards of these drugs on degradation potential of naturally occurring soil microflora. The prepared bacterial suspension was then introduced into the soil at a concentration of 1.6 × 10^7^ cells/g. Details on inoculum preparation are provided in the Supplementary materials. Throughout the experiment, soil moisture was maintained at 50% of the maximum water capacity. The study was conducted in the dark at 22 ± 1°C for 84 days. On days 0, 1, 7, 14, 28, 42, 56, 72, and 84, three random soil samples were collected for each treatment to determine NSAID concentrations.

### 2.5 Chemical analyses

To measure the concentrations of the tested NSAIDs in MSM medium samples, the procedure began with centrifugation to remove the proliferated MC-1/23 strain at 5,000 rpm for 5 min, followed by the addition of 0.1 mL of a 0.1% formic acid solution (v/v) to a 10 mL sample for acidification. Deionized water was then added to bring the final volume to 20 mL. The solution was extracted twice with 10 mL of ethyl acetate on a rotary shaker for 30 min. The resulting extracts were filtered through anhydrous Na_2_SO_4_ and evaporated to dryness at 45°C using a rotary evaporator (IKA, RV05 Basic, Janke & Kunkel-Ika Labortechnik, Germany). The dry residue was subsequently dissolved in 20 mL of a mixture of acetonitrile and 0.05% orthophosphoric acid solution (50:50, v/v) and analyzed chromatographically.

To determine NSAID concentrations in soil samples, the procedure began by adding 5 mL of a 0.1% formic acid solution (v/v) to a 10 g soil sample for acidification. Next, 15 mL of ethyl acetate was added, and the mixture was ultrasonicated for 10 min, following 5 min of shaking. The resulting suspension was centrifuged, and the supernatant was filtered through anhydrous Na_2_SO_4_. The residue was re-extracted with 15 mL of sodium acetate, followed by shaking, centrifugation, and filtration. The combined extracts were evaporated to dryness at 45°C using a rotary evaporator. The dry residue was dissolved in 20 mL of a mixture of acetonitrile and 0.05% orthophosphoric acid solution (50:50, v/v) and analyzed chromatographically. Details of the chromatographic system and conditions are provided in Table 3.

**Table 3:** Characterization of the chromatographic system and conditions used during the determination of the tested NSAIDs.

Parameter	Conditions/value
Chromatographic system	HPLC
Instrument	Shimadzu, Prominence-*i* LC-2030C 3D (Shimadzu, Inc., Japan)
Column	Luna 5 μm C18 100A, l = 250 mm, ϕ = 4.6 mm
Mobile phase	acetonitrile: 0.05% solution of ortho-phosphoric acid (70:30, *v*/*v*)
Temperature	40°C
Flow rate	1 mL/min
Volume of injection	20 μL
Wavelength	220 nm
Detection system	DAD
Retention time	NPX – 5 min, DCF – 10 min, IBF – 16 min
Data analysis	LabSolution LC-2030C 3D software

DAD, diode array detector; DCF, diclofenac; HPLC, high-performance liquid chromatography; IBF, ibuprofen; NPX, naproksen.

The analytical method used to quantify the tested NSAIDs was validated for linearity, specificity, precision, recovery, and limits of quantification and detection. The validation data are summarized in Table 4. Concentrations of test drugs in MSM and soil, presented in the results section, take into account the recoveries determined during the validation process. Calibration curves, chromatograms for NSAID standards, control and NSAID-treated MSM samples, as well as control and NSAID-treated soil samples obtained during the validation process, are depicted in Supplementary Figures S1–S6, respectively.

**Table 4:** Data obtained from the validation of the analytical method used to determine the concentrations of the tested NSAIDs in MSM and soil.

Parameter		Determined NSAIDs		
		IBF	DCF	NPX
Range of linearity		0.025–10.0 μg/mL, ie. 0.05–20.0 mg/L (kg)		
*R*^2^ for calibration curve		0.9994	0.9996	0.9999
LOQ		0.5 mg/L (kg)		
LOD		0.05 mg/L (kg)		
Recovery (%)/precision [RSD (%)] for MSM	LOQ	98.4/0.1	97.2/0.6	96.7/0.5
	10 × LOQ	99.2/0.2	98.1/0.3	97.9/0.1
Recovery (%)/precision [RSD (%)] for soil	LOQ	83.6/1.1	88.1/1.4	83.4/0.7
	10 × LOQ	97.5/0.1	93.0/0.2	86.9/0.1

DCF, diclofenac; IBF, ibuprofen; LOD, limit of detection; LOQ, limit of quantification; MSM, mineral salt medium; NPX, naproxen; RSD, relative standard deviation.

### 2.6 Data analysis

The dissipation of NSAIDs in MSM and soil was modeled using zero- or first-order kinetic equations. The rate constant *k* (day) for the zero-order reaction was determined using the equation *k* = (*C*_0_–*C*_t_)/*t*, while the equation *C*_t_/*C*_0_ = e*^–kt^* was used to determine *k* values for the first-order reaction, where *C*_0_ is the initial concentration of the drug in MSM or soil at time zero and *C*_t_ is the amount of drug in MSM or soil at time *t* (day). The half-life (*t*_1/2_) values, indicating the time required for NSAID concentrations in MSM or soil to decrease by half, were derived from the equations *t*_1/2_ = *C*_0_/2*k* or *t*_1/2_ = ln2/*k* for zero- or first-order kinetics, respectively. Data from three replicates per treatment were statistically analyzed using ANOVA. A three-way ANOVA was used to assess the level of variability (%) related to the factors studied, i.e., soil, drug and bacterial strain, and their interactions. *Post-hoc* comparisons of *t*_1/2_means were conducted using the least significant difference (LSD) test, with significance set at *p* < 0.05, employing the Statistica 13.3 PL software package.

## 3 Results

### 3.1 Identification of bacterial strain

**Figure 4:**
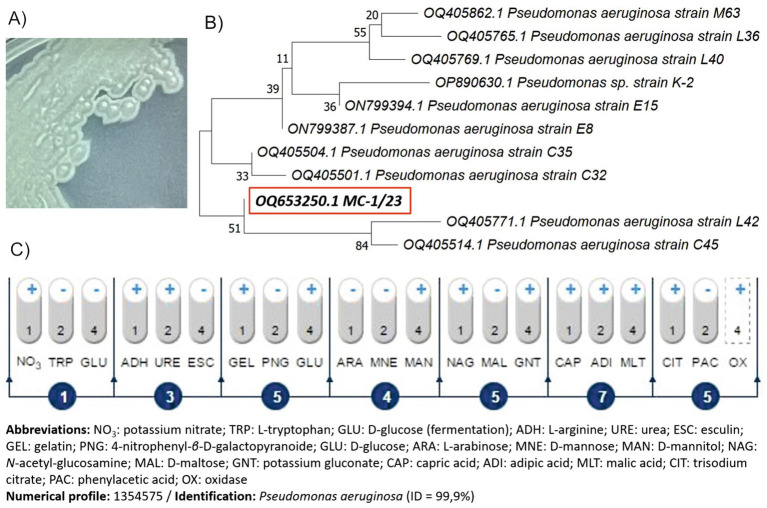
Colony appearance on nutrient agar **(A)**, phylogenetic tree based on the neighbor-joining method **(B)** and biochemical pattern based on the API 20 NE system **(C)** of the isolated strain MC-1/23. Bootstrap values from 1,000 replications are indicated at the branches. For each strain is given the GenBank accession number.

The screening process successfully isolated a bacterial strain from raw sewage in the presence of NSAIDs, which was designated as MC-1/23. The isolated strain belongs to bacteria that grow well on basic microbiological medium, i.e., nutrient agar, with characteristic green-blue colonies (Figure 4A). Analysis of the 16S rRNA gene sequence identified strain MC-1/23 as a member of the genus *Pseudomonas*, with high similarity to the species *P. aeruginosa* (Figure 4B). The sequence of strain MC-1/23 has been deposited in GenBank under the accession number OQ653250 (Supplementary Figure S7). Additionally, biochemical testing confirmed the strain’s identity as *P. aeruginosa* with 99.9% accuracy (Figure 4C).

### 3.2 Degradation of NSAIDs in liquid medium

#### 3.2.1 Degradation of ibuprofen

**Figure 5:**
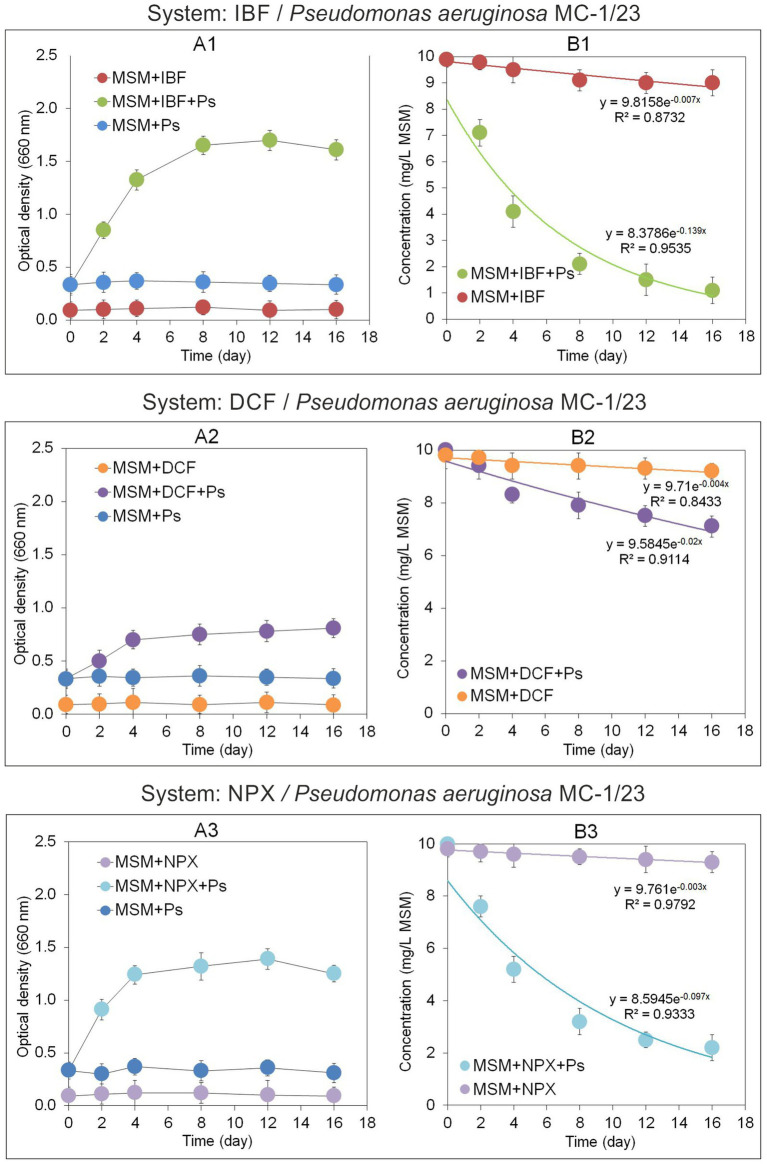
Growth of the bacterial strain MC-2/23 **(A1–A3)** and regression curves obtained from the concentrations of the analyzed NSAIDs **(B1–B3)** in the degradation experiment with mineral salt medium. The data presented are the means of three replicates. Bars represent standard deviations, which were within 5% of the means (MSM, mineral salt medium; IBF, ibuprofen; DCF, diclofenac; NPX, naproxen; Ps, *Pseudomonas aeruginosa* strain MC-1/23).

**Figure 6:**
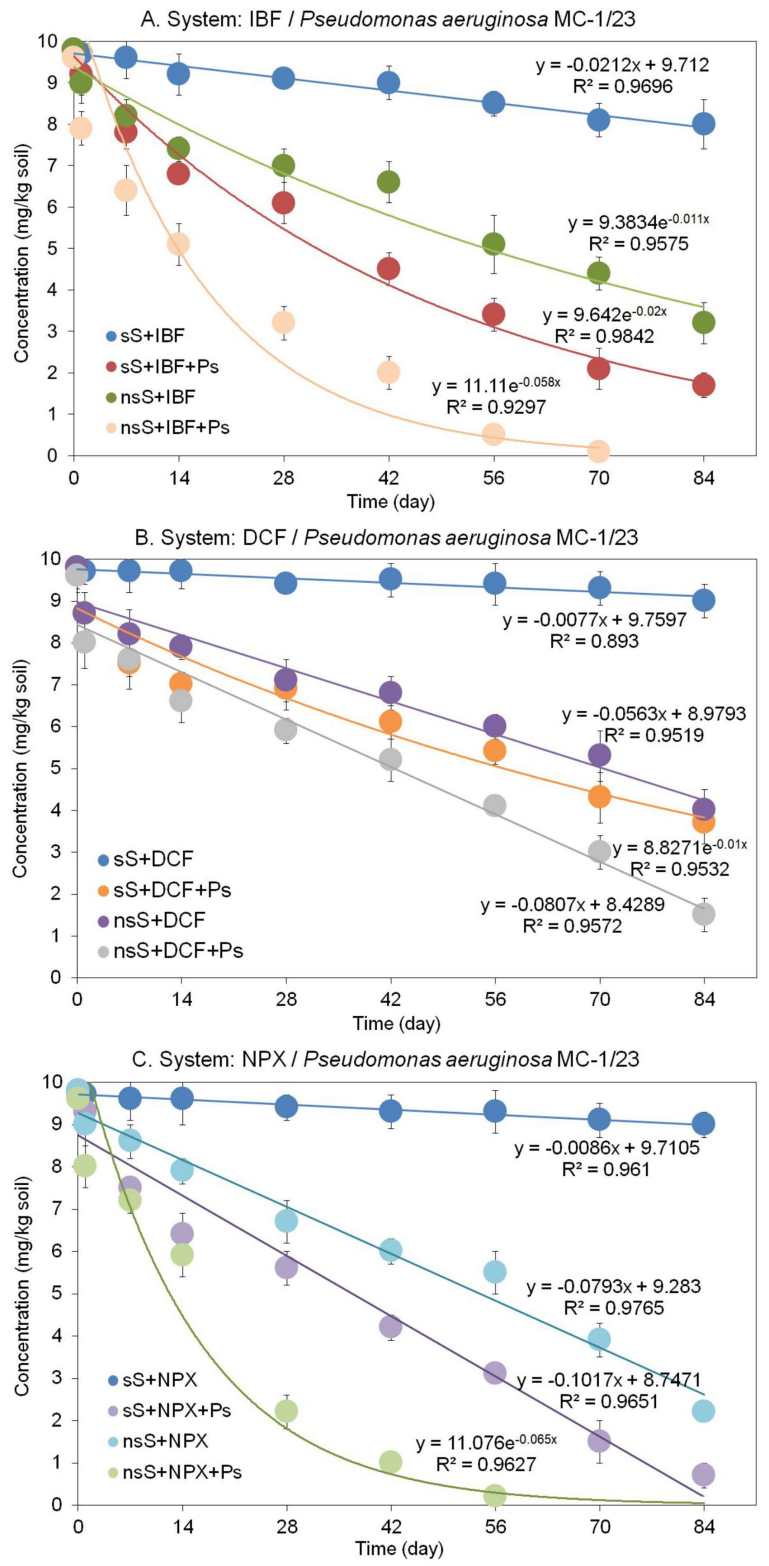
Concentrations and regression curves obtained for ibuprofen **(A)**, diclofenac **(B)**, and naproxen **(C)** in the degradation experiment with soil. The data presented are the means of three replicates. Bars represent standard deviations, which were within 5% of the means (sS, sterile soil; nsS, nonsterile soil; IBF, ibuprofen; DCF, diclofenac; NPX, naproxen; Ps, *Pseudomonas aeruginosa* strain MC-1/23).

The growth dynamics of the *P. aeruginosa* MC-1/23 strain in MSM were assessed by measuring absorbance at OD 660 nm, as shown in Figure 5A1. The most pronounced growth of the strain in IBF-supplemented medium (MSM + IBF + Ps) was observed on the 12th day of incubation, after which the culture reached the stationary phase. By contrast, no changes in optical density were observed in the control samples (MSM + Ps and MSM + IBF) during the 16-day incubation period (Figure 5A1). Bacterial growth in MSM supplemented with IBF corresponded with the drug’s degradation rate (Figure 5B1). The strain degraded 89% of the initial IBF dose in MSM + IBF + Ps within 16 days of incubation. Regression analysis and kinetic modeling (Figure 6A) indicated that the degradation process in MSM + IBF + Ps was characterized by a rate constant (*k*) of 0.139/day, with a *t*_1/2_value of 5.0 days (Table 5). By comparison, in the control without bacterial inoculum (MSM + IBF), IBF underwent minimal degradation, with 91% of the initial drug dose remaining at the end of the experiment (Figure 5B1).

**Table 5:** Degradation parameters for the analyzed NSAIDs obtained from the experiment with mineral salt medium.

Treatment	Kinetic model	*k* (day)	*t*_1/2_ (day)
MSM + IBF + Ps	First-order reaction	0.139 ± 0.006	5.0 ± 0.2^c^
MSM + DCF + Ps	First-order reaction	0.020 ± 0.001	34.7 ± 1.7^a^
MSM + NPX + Ps	First-order reaction	0.097 ± 0.004	7.1 ± 0.3^b^

MSM, mineral salt medium; IBF, ibuprofen; DCF, diclofenac; NPX, naproxen; Ps, *Pseudomonas aeruginosa* strain MC-1/23; k, degradation rate constant obtained from the regression equation shown in Figures 5B1–B3; *t*_1/2_, half-life calculated from the equation *t*_1/2_ = ln2/*k*. The data presented are the means of three replicates ± standard deviation, which was within 5% of the mean. Significant differences (LSD test, *p* < 0.05) between the *t*_1/2_ values for treatments are marked with different letters.

#### 3.2.2 Degradation of diclofenac

The growth dynamics of the MC-1/23 strain in MSM are illustrated in Figure 5A2. The highest value of the culture optical density in DCF-supplemented medium (MSM + DCF + Ps) was observed after 16 days of incubation. By contrast, no changes in optical density were noted for the controls (MSM + Ps and MSM + DCF), with both remaining stable throughout the incubation period. Experimental data revealed that *P. aeruginosa* (MSM + DCF + Ps) degraded 29% of the initial DCF dose over 16 days of incubation (Figure 5B2). Regression analysis and kinetic modeling (Figure 5B2) indicated a degradation rate constant (*k*) of 0.020/day, with a *t*_1/2_value of 34.7 days (Table 5). By comparison, the abiotic control (MSM + DCF) exhibited minimal DCF degradation, with only 2% of the initial dose degraded (Figure 5B2).

#### 3.2.3 Degradation of naproxen

The growth dynamics of the MC-1/23 strain in MSM are shown in Figure 5A3. The strain exhibited its highest growth rate in NPX-supplemented medium (MSM + NPX + Ps) after 12 days of incubation. By contrast, the controls (MSM + Ps and MSM + NPX) showed no changes in optical density, remaining constant throughout the incubation period. Experimental results indicated that *P. aeruginosa* (MSM + NPX + Ps) degraded 78% of the initial NPX dose within 16 days of incubation (Figure 5B3). Regression analysis and kinetic modeling (Figure 5B3) revealed a degradation rate constant (*k*) of 0.097/day and a *t*_1/2_value of 7.1 days (Table 5). In comparison, the abiotic control (MSM + NPX) showed minimal degradation of NPX, with only 2% of the initial dose degraded (Figure 5B3).

### 3.3 Degradation of NSAIDs in soil

#### 3.3.1 Degradation of ibuprofen

The degradation of IBF in sterile soil inoculated with *P. aeruginosa* MC-1/23 (sS + IBF + Ps) was described by a rate constant (*k*) of 0.021/day (Table 6). After 84 days of incubation, 82.7% of the initial IBF concentration was degraded (Figure 6A), and the *t*_1/2_value, representing the time required for the IBF concentration to decrease by half, was calculated to be 34.9 days (Table 6). In nonsterile soil inoculated with the bacterial strain (nsS + IBF + Ps), the degradation was more rapid, with a higher rate constant (*k*) of 0.058/day and a lower *t*_1/2_value of 12.0 days (Table 6). By contrast, IBF degradation in nonsterile soil without the bacterial strain (nsS + IBF) had a rate constant (*k*) of 0.011/day and a *t*_1/2_value of 63.4 days (Table 6), with 67.3% of the initial concentration degraded after 84 days (Figure 6A). The abiotic control exhibited a much slower degradation process, characterized by a rate constant (*k*) of 0.021/day (Table 6). At the end of the incubation period, 81.6% of the initial drug dose remained in the abiotic control (Figure 6A).

**Table 6:** Degradation parameters for the analyzed NSAIDs obtained from the experiment with soil.

System	Treatment	Kinetic model	*k* (day)	*t*_1/2_ (day)
IBF/Ps	sS + IBF	Zero-order reaction	0.021 ± 0.003	237.3 ± 35.7^Ba^
	sS + IBF + Ps	First-order reaction	0.020 ± 0.002	34.9 ± 3.5^Cc^
	nsS + IBF	First-order reaction	0.011 ± 0.001	63.4 ± 5.8^Bb^
	nsS + IBF + Ps	First-order reaction	0.058 ± 0.004	12.0 ± 0.8^Bd^
DCF/Ps	sS + DCF	Zero-order reaction	0.008 ± 0.001	619.0 ± 78.0^Aa^
	sS + DCF + Ps	First-order reaction	0.010 ± 0.001	69.8 ± 3.0^Ac^
	nsS + DCF	Zero-order reaction	0.056 ± 0.004	87.8 ± 2.6^Ab^
	nsS + DCF + Ps	Zero-order reaction	0.081 ± 0.005	60.7 ± 1.1^Ad^
NPX/Ps	sS + NPX	Zero-order reaction	0.009 ± 0.001	549.0 ± 61.4^Aa^
	sS + NPX + Ps	Zero-order reaction	0.102 ± 0.007	48.2 ± 3.2^Bc^
	nsS + NPX	Zero-order reaction	0.079 ± 0.007	62.4 ± 2.8^Bb^
	nsS + NPX + Ps	First-order reaction	0.065 ± 0.005	10.7 ± 0.7^Bd^

sS, sterile soil; nsS, non-sterile soil; IBF, ibuprofen; DCF, diclofenac; NPX, naproxen; Ps, *Pseudomonas aeruginosa* strain MC-1/23; k, degradation rate constant obtained from the regression equation shown in Figures 6A–C; *t*_1/2_, half-life calculated from the equations *t*_1/2_ = C_0_/2*k* or *t*_1/2_ = ln2/*k* for zero- or first-order kinetics. The data presented are the means of three replicates ± standard deviation, which was within 5% of the mean. Significant differences (LSD test, *p* < 0.05) for *t*_1/2_ between treatments within one system and between treatments from various systems are denoted with different lower and uppercase letters, respectively.

#### 3.3.2 Degradation of diclofenac

The results demonstrated that DCF degradation dynamics in soil varied depending on the conditions and the presence of the bacterial strain (Figure 6B). The slowest degradation rate was observed in sterile soil (sS + DCF), with only 8.2% of the initial DCF concentration degraded after 84 days. This process was characterized by a rate constant (*k*) of 0.008/day (Table 6). In sterile soil inoculated with *Pseudomonas aeruginosa* MC-1/23 (sS + DCF + Ps), degradation occurred more rapidly than in sterile soil without inoculation, achieving 62.2% degradation by the end of the incubation period (Figure 6B). The *t*_1/2_value, representing the time for the DCF concentration to decrease by half, was 69.8 days, which was 8.9 times lower than in sterile soil without inoculum (Table 6). In nonsterile soil (nsS), the results indicated that the indigenous microflora exhibited degradation activity. DCF was degraded at a rate of 59% over the 84-day experiment (Figure 6B), with a rate constant (*k*) of 0.056/day and a *t*_1/2_value of 87.8 days (Table 6). When nonsterile soil was inoculated with *P. aeruginosa* MC-1/23 (nsS + DCF + Ps), the degradation rate was further accelerated, resulting in 84% degradation of the initial drug concentration (Figure 6B). The degradation process in this condition was characterized by a rate constant (*k*) of 0.081/day and a *t*_1/2_value of 60.7 days (Table 6).

#### 3.3.3 Degradation of naproxen

Similar to the other drugs tested, NPX exhibited varying degradation dynamics depending on the conditions (Figure 6C). The slowest degradation rate was observed in sterile soil (sS), where only 8% of the initial NPX concentration was degraded after 84 days. This process was characterized by a rate constant (*k*) of 0.009/day (Table 6). In sterile soil inoculated with *P. aeruginosa* MC-1/23 (sS + NPX + Ps), degradation occurred more rapidly, achieving 93% degradation of the initial concentration by the end of the incubation period (Figure 6C), with a *t*_1/2_value of 48.2 days (Table 6). In nonsterile soil (nsS + NPX), the results indicated that the native microflora had a significant ability to degrade NPX. Within 84 days, 78% of the drug was degraded (Figure 6C), with a rate constant (*k*) of 0.079/day and a *t*_1/2_value of 62.4 days (Table 6). Inoculating nonsterile soil with *P. aeruginosa* MC-1/23 (nsS + NPX + Ps) further accelerated NPX degradation, resulting in complete (100%) degradation of the initial concentration. Under these conditions, the degradation process was characterized by a rate constant (*k*) of 0.065/day and a *t*_1/2_value of 10.7 days (Table 6).

## 4 Discussion

The soil ecosystem, driven by microbial activity, exhibits a specific potential to mitigate NSAID pollution by degrading these compounds, albeit to a limited extent (Topp et al., 2008; Xu et al., 2009; Carr et al., 2011; Aznar et al., 2014; Cycoń et al., 2017; Muter, 2023). Various methods are currently employed to remediate soil contamination, with bioaugmentation—a “green technology”—playing a significant role in removing organic pollutants. Bioaugmentation is particularly useful in locations where indigenous microorganisms are either insufficient in number or lack the metabolic capacity to degrade contaminants (Cycoń et al., 2017). In soils, bioaugmentation enhances the catabolic potential of the microbial community through the introduction of specific bacterial or fungal strains, or consortia thereof, with targeted catabolic capabilities (Singer et al., 2005; Cycoń et al., 2017; Muter, 2023). However, their introduction into soils via *in situ* bioaugmentation may be limited by their incapability to demonstrate their contaminant-degrading activity as under laboratory conditions. As has been shown, there may be a number of reasons related to this phenomenon, one of which may be the ability of degraders to switch into a viable but non-culturable (VBNC) or dormant state with low metabolic activity after introduction into natural environmental matrix (Santos et al., 2019; Han et al., 2023; Zhou et al., 2023; Papin et al., 2024). In this study, a bacterial strain with NSAID-degrading ability was isolated. Enriched cultivation techniques facilitated the isolation of strain MC-1/23, identified as *P. aeruginosa*, which demonstrated a specific degradation potential for IBF, DCF, and NPX. The strain was capable of utilizing these compounds as carbon and energy sources in mineral salt medium, suggesting metabolic degradation. However, the degradation rates varied among the drugs. IBF showed the highest degradation rate (89%, *t*_1/2_ = 5.0 days), followed by NPX (78%, *t*_1/2_ = 7.1 days), while DCF exhibited the slowest degradation (29%, *t*_1/2_ = 34.7 days). These variations in degradation levels are likely attributable to differences in the chemical structures and physicochemical properties of the drugs.

Bacteria from the genus *Pseudomonas*, known for their high metabolic activity, exhibit a broad spectrum of biochemical capabilities that enable them to degrade various natural and synthetic compounds (Ahmed et al., 2001; Nelson et al., 2002). These bacteria have been isolated from diverse environments contaminated with a range of chemicals (Pacwa-Płociniczak et al., 2014; Żur et al., 2020; Poddar et al., 2024). However, the literature lacks reports of *P. aeruginosa* strains capable of degrading all three NSAIDs tested in this study – IBF, DCF, and NPX. Nevertheless, earlier studies demonstrated that certain strains of this species have a strong potential for IBF degradation. For instance, the *P. aeruginosa* S1 strain utilized IBF as the sole carbon and energy source in a mineral medium, reducing the initial concentration of 40 mg/L by 38 and 75% after 3 and 6 days, respectively (Mohamed et al., 2023). Similarly, Liu et al. (2023) reported that *P. aeruginosa* strain LY.1 could grow in a medium containing flurbiprofen as the sole carbon source. Under optimal conditions (400 mg/L initial flurbiprofen concentration, 30°C, and pH 7), this strain achieved complete degradation of the drug within 96 h. Other studies have highlighted the degradation capabilities of different *Pseudomonas* species. Żur et al. (2020) demonstrated that *Pseudomonas moorei* strain KB4 degraded DCF in a monosubstrate culture at a concentration of 0.5 mg/L, with glucose and sodium acetate supplementation enhancing degradation to 1 mg/L within 12 days. Poddar et al. (2022) investigated *Pseudomonas* sp. strain PrS10, which exhibited a high degradation potential for paracetamol, achieving a 92.35% degradation efficiency as confirmed by GC–MS analysis. However, the addition of carbohydrates to the culture medium reduced degradation efficiency due to competition between monosaccharides and paracetamol as energy sources. Other bacterial and fungal species have also shown varying degradation capabilities. For example, *Bacillus thuringiensis* B1 degraded 5 mg/L of IBF within 2 days but showed minimal degradation of NPX, likely due to insufficient carbon and energy sources provided by the drugs (Marchlewicz et al., 2016). By contrast, *Sphingomonas* Ibu-2 and Var*iovorax* Ibu-1 strains were capable of degrading IBF at high concentrations (Murdoch and Hay, 2015). Additionally, *Stenotrophomonas maltophila* strain KB2 degraded 78% of NPX in the presence of glucose (Wojcieszyńska et al., 2014), while exposure of *Klebsiella* sp. strain KSC to DCF at a concentration of 70 mg/L resulted in complete mineralization within 72 h (Stylianou et al., 2018). Fungal species have also demonstrated significant degradation potential. Marco-Urrea et al. (2010) reported that *Trametes versicolor* transformed 94% of DCF (10 mg/L) and adsorbed 47% of the drug onto fungal cell surfaces. Similarly, *Phanerochaete chrysosporium* transformed 93% of DCF within 30 days, highlighting its ability to degrade polycyclic NSAIDs effectively (Rodarte-Morales et al., 2012).

As shown by many studies, laccase, catechol 1,2-dioxygenase, catechol 2,3-dioxygenase, catechol 4,5-dioxygenase, and peroxidase are involved in microbial biotransformation of IBF (Murdoch and Hay, 2015; Marchlewicz et al., 2017; Fetyan et al., 2024). Moreover, enzymes including cytochrome p450 system monooxygenase, glucuronidase, dioxygenases, and hydrolases, among others, have been found to be responsible for the degradation of DCF (Żur et al., 2020; Żur et al., 2021; Wojcieszyńska et al., 2023; Poddar et al., 2024), whereas monooxygenase, naphthalene dioxygenase, hydroxyquinol 1,2-dioxygenase, gentisate 1,2-dioxygenase, protocatechuate 3,4-dioxygenase and protocatechuate 4,5-dioxyegnase are involved in the microbial degradation of NPX (Wojcieszyńska et al., 2014; Dzionek et al., 2024). Many of these enzymes, involved in the degradation of discussed NSAIDs, have been found in strains belonging to *P. aeruginosa* capable of degrading other chemicals, especially aromatic compounds (Chebbi et al., 2017; Muthukumar et al., 2022; Hossain et al., 2024; Li et al., 2024). In our study, we did not determine the formed metabolites, and the degradation potential of the MC-1/23 strain was assessed by the decrease in concentrations of parent compounds during incubation. However, based on chemical structures of IBF, DCF and NPX, as well as the findings of other studies, it is reasonable to assume that the same enzymes may be involved during the degradation of these drugs and similar degradation products may be formed, as has been shown for the degradation of aromatic compounds using strains belonging to *P. aeruginosa* and the degradation of NSAIDs proceeded by other microorganisms.

The results revealed varying dynamics in the degradation of the tested NSAIDs in soil, influenced by both the experimental conditions and the bacterial strain used. In general, pharmaceuticals in soil undergo a range of physical, chemical, and biochemical processes (Chefetz et al., 2008; Monteiro and Boxall, 2010; Lin et al., 2023). The fate and persistence of individual NSAIDs in soil depend on factors such as the physicochemical properties of the compound and environmental characteristics (Topp et al., 2008; Lin et al., 2023; Shu et al., 2023). This study demonstrated that IBF exhibited the highest susceptibility to degradation, while DCF showed the lowest, irrespective of the conditions applied. The slowest degradation rates for all tested NSAIDs were observed in sterile soil. The study also indicated that the natural soil microflora was capable of degrading the introduced drugs at a concentration of 10 mg/kg soil, with *t*_1/2_values ranging from 62.4 days for NPX to 87.8 days for DCF. Previous studies also confirmed the role of microorganisms in NSAID degradation in soil. For instance, Al-Rajab et al. (2010) reported that DCF was rapidly mineralized in soils of different textures, with a half-life of <5 days. Similarly, other research found that NPX, DCF, and IBF exhibited relatively fast degradation rates in soil, with maximum half-lives of 20.44 days, although IBF degraded more slowly than the others (Xu et al., 2009). Shu et al. (2021) observed low to moderate persistence for IBF and NPX in soil, with half-lives ranging from 4.9 to 14.8 days, whereas KTP displayed higher persistence, with an average half-life of 33 days. Additionally, earlier studies highlighted the concentration-dependent degradation of NSAIDs in nonsterile soil. DCF, IBF, and NPX at a concentration of 0.05 mg/kg soil degraded rapidly, with half-lives ranging from 0.2 to 9.5 days. However, at a concentration of 5 mg/kg soil, degradation rates significantly decreased, extending half-lives to as much as 68 days (Grossberger et al., 2014). Xu et al. (2009) also reported that increasing initial soil concentrations of NPX, DCF, and IBF reduced their degradation rates, suggesting microbial activity was inhibited at higher drug levels. Previous studies have indicated that high doses of NSAIDs may adversely affect microbial structure and activity in soil. For example, Cycoń et al. (2016) found an inhibitory effect of ibuprofen, diclofenac, and naproxen at a dose of 10 mg/kg soil on substrate-induced respiration, dehydrogenases, and phosphatases as well as on the abundance of cultured bacteria and fungi. However, the effect was observed only at the beginning of the 90-day study, while during the experiment, the number of heterotrophic bacteria and fungi, as well as respiratory and enzymatic activity increased significantly, which might be a consequence of the evolution of specific microorganisms capable of degrading NSAIDs and can be used as additional sources of carbon and energy. Also, Kovacs et al. (2024) used the phospholipid-derived fatty acid (PLFA) approach and observed a decrease in the abundance of the soil microbiome (especially fungi) in the tested soils in response to the exposure to ibuprofen and diclofenac. Although the authors observed signs of resistance for bacteria at the end of the experiment, the bacterial community abundance was not similar to that observed at the beginning of the study. By contrast, in our study, no lag phase was observed in the degradation of tested drugs at the concentration of 10 mg/kg soil, suggesting that the applied drugs did not significantly suppress the activity of indigenous microorganisms.

The study demonstrated that *P. aeruginosa* MC-1/23 could degrade all tested NSAIDs in sterile soil, although the degradation rates varied, with *t*_1/2_values of 34.9, 69.8, and 48.2 days for IBF, DCF, and NPX, respectively. Overall, strain MC-1/23 exhibited a higher degradation potential for NSAID removal in sterile soil compared with the natural microflora in nonsterile soil. Notably, no lag phases were observed, likely because the inoculated strain had prior exposure to NSAIDs, enabling faster adaptation to these compounds than the indigenous soil microflora, which had no previous contact with NSAIDs. A similar phenomenon has been reported in studies on the degradation of other contaminants (Abo-Amer, 2011; Chen et al., 2014; Aceves-Diez et al., 2015; Nwankwegu et al., 2022). Bioaugmentation of nonsterile soil with *P. aeruginosa* MC-1/23 significantly enhanced the degradation of the tested drugs, reducing *t*_1/2_values 5.3-fold, 1.4-fold, and 5.8-fold for IBF, DCF, and NPX, respectively, compared with soils with only natural microflora. This finding confirms that the introduced strain increased the catabolic potential of the indigenous microorganisms. In general, statistical analysis showed a significant effect (*p* < 0.001) of the *P. aeruginosa* MC-1/23 strain on the degradation rate of all tested drugs in soil, confirming its degradation and bioremediation potential, and the strain effect explained most of the variance observed (33%) (Table 7). The significant effect of soil (*p* < 0.001) and drug (*p* < 0.001) on degradation have also been demonstrated and these factors accounted for 27 and 6% of variance observed, respectively. In addition, interactions between the factors tested also had a significant effect (*p* < 0.001) on the rate of degradation, with the interaction between soil and bacterial strain accounting for the largest contribution (21%) to the variability observed (Table 7).

**Table 7:** Results of the three-way ANOVA for *t*_1/2_ values obtained in the experiment with soil.

Source of variation	*df*	SS	MS	Variance explained (%)	*F*	*P*
Soil (S)	1	397627.8	397627.8	27	421.3433	<0.001
Drug (D)	2	92989.5	46494.8	6	49.2678	<0.001
Bacterial strain (Bs)	1	477896.2	477896.2	33	506.3991	<0.001
S*D	2	56328.3	28164.1	4	29.8439	<0.001
S*Bs	1	314822.5	314822.5	21	333.5992	<0.001
D*Bs	2	48474.1	24237.1	3	25.6826	<0.001
S*D*Bs	2	57964.6	28982.3	4	30.7109	<0.001

In this study, an inoculum density of 1.6 × 10^7^ cells/g soil proved sufficient for the degradation of the tested NSAIDs. Previous research has shown that inoculum size is a critical factor in the effective biodegradation of various soil contaminants, including antibiotics, pesticides, and petroleum compounds (Bidlan et al., 2004; Chen et al., 2014; Leng et al., 2016; Wen et al., 2018). For example, an inoculum density below 10^4^ cells/g soil was found to be ineffective in pesticide degradation due to the low survival rates of the introduced bacteria. Singh et al. (2006) reported that *Enterobacter* sp. failed to degrade the pesticide chlorpyrifos when introduced at an inoculum density of 10^3^ cells/g soil. This might be due to a rapid decline in the number of introduced strains in the soil caused by various environmental factors, such as biological related to the presence of other microorganisms and physicochemical including pH, moisture content, temperature, food availability, or toxic compounds (Kamjumphol et al., 2015; Fida et al., 2017; O’Callaghan et al., 2022; Han et al., 2023; Papin et al., 2024). Thus, using an inoculum of adequate density can offset the initial decline in bacterial population, ensuring sufficient survivors to participate in contaminant degradation (Comeau et al., 1993; Duquenne et al., 1996). Although the survival ability of the introduced MC-1/23 strain into soil was not directly monitored in the present study, it can be concluded indirectly from the results that the introduced strain had survival capabilities. For all drugs tested, in both sterile and non-sterile soil, the MC-1/23 strain showed higher degradation potential compared to non-sterile soil with only natural microflora. In our study, no lag phase was observed in the degradation of tested drugs and the inoculum size of 1.6 × 10^7^ cells/g of soil seems to be adequate for this process.

The degradation of the tested NSAIDs in nonsterile soil may be influenced not only by the characteristics of the soil’s microbial community but also by its physicochemical properties. The soil used in this study was classified as sandy loam with a high sand fraction and low organic matter content. These properties likely reduced the affinity of NSAIDs for soil particles, increasing their availability to microorganisms responsible for degradation (Xu et al., 2010; Vasiliadou et al., 2013; Graouer-Bacart et al., 2016; Lin et al., 2023). Chefetz et al. (2008) demonstrated that both the quantity and physicochemical nature of soil organic matter significantly impact the sorption of DCF and NPX. Similarly, Shu et al. (2021) found that the degradation of NPX, KTP, and IBF in soils supplemented with alkaline-treated biosolids was inhibited compared with untreated soils. Conversely, the addition of biosolids to sterile or nonsterile soil did not enhance the dissipation of DCF, as also noted by Al-Rajab et al. (2010). This phenomenon is consistent with observations for other pharmaceuticals (Topp et al., 2008; Al-Rajab et al., 2015; Jauregi et al., 2023; Nkoh et al., 2024) and organic contaminants, including polycyclic aromatic hydrocarbons (Chen and Yuan, 2011; Zhang et al., 2023; Omoni et al., 2024) and pesticides (Karpouzas and Walker, 2000; Cycoń et al., 2014; Cervantes-Díaz et al., 2024; Sun et al., 2024). The degradation rates of the tested NSAIDs may also be attributed to their chemical structures and physicochemical properties, which determine their toxicity to soil microorganisms (Dastidar et al., 2000; Lawrence et al., 2007; Lin et al., 2023) and their susceptibility to degradation (Xu et al., 2009; Lin and Gan, 2011; Domaradzka et al., 2015; Shu et al., 2023). The low solubility of NSAIDs may limit their bioavailability to microorganisms, thereby reducing their biodegradation potential and contributing to their persistence in soil (Xu et al., 2009; Al-Rajab et al., 2015; Lin et al., 2023). In the present study, diclofenac sodium showed the slowest degradation rate despite the highest solubility rate (14 mg/mL) compared to the other drugs tested (Table 1), indicating its maximal availability to microorganisms. This finding could be ascribed to the formation of diclofenac post-hydrolysis of diclofenac sodium and its low water solubility at 2.4 mg/L (Wojcieszyńska et al., 2023). This feature of DCF, combined with the presence of a chlorinated ring in its molecule, may result in a high resistance to biodegradation processes. By contrast, IBF and NPX, with a significantly higher solubility of 21 and 15.9 mg/L, respectively, showed faster degradation rates. DCF generally exhibit a higher affinity for soil organic matter, resulting in reduced degradation rates and longer persistence in the soil environment (Chefetz et al., 2008; Dolu and Nas, 2023; Shu et al., 2023).

The findings of this study are challenging to compare with those of other researchers due to the lack of prior investigations into the use of bacteria for the bioremediation of NSAID-contaminated soils. This is the first time that bioaugmentation with bacteria belonging to *P. aeruginosa* has been used for degradation of IBF, DCF and NPX in soil. Nonetheless, earlier studies have demonstrated the effectiveness of bioaugmentation in remediating soils contaminated with antibiotics, pesticides, and aromatic hydrocarbons (Yang and Lee, 2007; Anwar et al., 2009; Nwankwegu et al., 2022; Muter, 2023). Despite this potential, employing bacteria to degrade NSAIDs and other organic pollutants in contaminated soils presents certain challenges, particularly given the limited understanding of the fate of introduced inoculants in the soil. The survival and competitiveness of the inoculants against native microorganisms, as well as their degradative activity, are critical factors that may constrain the success of bioremediation efforts (Yang and Lee, 2007; Anwar et al., 2009).

## 5 Conclusion

The screening procedure employing enriched cultivation enabled the isolation of the bacterial strain MC-1/23, identified as *P. aeruginosa*, which demonstrated a specific degradation potential for the selected NSAIDs: IBF, DCF, and NPX. In this study, strain MC-1/23 utilized these compounds in mineral salt medium as carbon and energy sources, indicating their potential for metabolic degradation. The results revealed varying degradation dynamics for the tested NSAIDs in soil, influenced by both the experimental conditions and the bacterial strain. Among the drugs tested, IBF showed the highest susceptibility to degradation, while DCF exhibited the lowest, regardless of the conditions applied. These differences likely stem from the distinct chemical structures and physicochemical properties of the drugs. The study confirmed that both abiotic conditions and biotic factors contribute to the degradation of NSAIDs in soil, with microbial activity playing the predominant role. Furthermore, bioaugmentation of nonsterile soil with *P. aeruginosa* strain MC-1/23 significantly enhanced the degradation rates of the drugs. *T*_1/2_values decreased by 5.3-, 1.4-, and 5.8-fold for IBF, DCF, and NPX, respectively, compared with soils containing only natural microflora. This improvement demonstrates that the introduced strain increased the catabolic potential of the native microbial community. The degradation and bioremediation capabilities of strain MC-1/23 for all tested NSAIDs highlight its potential application in the remediation of contaminated soils. However, before the strain can be used in large-scale bioremediation, further research is needed on the molecular and biochemical aspects of NSAID degradation by the MC-1/23 strain to establish the exact pathways for their degradation, accurately assess the survival of the strain and its competitiveness with native microflora to understand the strain’s interaction with the soil environment.

## Data Availability

The data presented in the study are deposited in the National Center for Biotechnology Information (NCBI) repository, with accession number OQ653250.
