# Supplementary material for: Potential of newly isolated strain Pseudomonas aeruginosa MC-1/23 for the bioremediation of soil contaminated with selected non-steroidal anti-inflammatory drugs
Source: Front Microbiol. 2025 Mar 3;16:1542875. doi: 10.3389/fmicb.2025.1542875 (PMC11912566; doi:10.3389/fmicb.2025.1542875)
Supplement: Supplementary file 1 [file Data_Sheet_1.PDF]

SUPPLEMENTARY MATERIALS

Potential of newly isolated strain *Pseudomonas aeruginosa* MC-1/23 for the bioremediation of soil contaminated with selected non-steroidal anti-inflammatory drugs (NSAIDs)

Magdalena Klim [1], Agnieszka Żmijowska [2], Mariusz Cycoń [1,*]

[1] Department of Microbiology, Faculty of Pharmaceutical Sciences, Medical University of Silesia, Jagiellońska 4, 41-200 Sosnowiec, Poland;
[2] Laboratory of Analytical Chemistry, Ecotoxicology Research Group, Łukasiewicz Research Network - Institute of Industrial Organic Chemistry Branch Pszczyna, Doświadczalna 27, 43-200 Pszczyna, Poland;

*** Correspondence:**
Mariusz Cycoń
mcycon@sum.edu.pl

Materials and methods

Isolation procedure of bacterial strain

The bacterial strain capable of degrading the selected drugs was isolated from raw sewage obtained from the municipal sewage treatment plant "Gigablok" in Katowice-Szopienice, southern Poland. The isolation process employed a two-step enrichment procedure using a mineral salt medium (MSM), the composition of which is shown in Supplementary Table S2. The initial stage of isolation involved enriched cultivation in MSM supplemented with a mixture of commercially available NSAIDs. To achieve this, 10 mL of raw sewage was added to 300 mL flasks containing 100 mL of MSM enriched with Ibuprom, Diclac Duo 150, and NPX 500. The drugs were added to achieve a final concentration of 10 mg/L for each active ingredient (IBF, DCF, and NPX) in the medium. The samples were incubated for 96 hours on a rotary shaker at 120 rpm in a darkened thermostatic chamber maintained at 30°C ± 1°C. Subsequently, 1 mL of the suspension was transferred to flasks containing fresh MSM with the same concentration of NSAIDs and incubated under identical conditions for an additional 96 hours. This transfer process was repeated seven times. In the second phase of isolation, the procedure was conducted using MSM supplemented with reference standards of the tested drugs. Specifically, 1 mL of suspension from the previous stage was added to 300 mL flasks containing 100 mL of MSM with IBF, DCF, and NPX at a concentration of 10 mg/L each. The samples were incubated for 96 hours under the same conditions as in the first stage, followed by another series of seven transfers into fresh

medium. Afterward, serial dilutions of the flask samples were plated onto MSM agar plates supplemented with IBF, DCF, and NPX (10 mg/L each) to isolate individual colonies. These plates were incubated for 96 hours in a darkened thermostatic chamber at 30°C ± 1°C. Distinct colonies were purified by repeated streaking on the same agar medium. Ultimately, a strain capable of growing on a mineral medium containing each of the tested drugs was successfully selected.

Preparation procedure of bacterial inoculum

The bacterial strain was cultured in 200 mL Erlenmeyer flasks containing 100 mL of nutrient broth (BTL, Poland). At the exponential phase, the bacteria were pelleted by centrifugation (5 min, 10,000 g). The pellet was washed twice with 0.85% of sterile NaCl and then resuspended in 0.85% of sterile NaCl to obtain a bacterial suspension at a concentration of approximately 2.1×10^9 cells/mL. The cell density (OD 660 nm) was measured using a densitometer (Densimat®, bioMérieux, France). Next, the bacterial suspension was introduced into MSM or soil in order to produce a final bacterial count of approximately 1.6×10^7 cells/mL MSM or g soil.

Table S1. Composition of commercially available NSAIDs used in the experiment.

Pharmaceutical preparation	Contents
Ibuprom	ibuprofen 200 mg, cellulose, corn starch, guar gum, talc, crospovidone Type A, silica, colloidal hydrate, *hydrogenated vegetable oil, hydroxypropyl cellulose,* macrogol 400, gelatin, sucrose, caolin, confectioners sugar, calcium carbonate, acacia gum, titanium dioxide E171, Opalux White AS 7000, Carnauba wax, Opacode Black S-1-17823
Diclac® Duo 150	diclofenac sodium salt 150 mg, lactose monohydrate, calcium hydrogen phosphate dehydrate, microcrystalline cellulose, magnesium stearate, carboxymethyl starch sodium salt Type A, silica colloidal anhydrous, corn starch, iron oxide E172, hypromellose
Naproxen 500	naproxen 500 mg, methyl cellulose, croscarmellose sodium, magnesium stearate, silica colloidal anhydrous

Table S2. Composition of mineral salt medium (MSM).

Component	Amount [g/L]
$(NH_4)_2SO_4$	2.0
$MgSO_4 \cdot 7H_2O$	0.2
$CaCl_2 \cdot 2H_2O$	0.01
$FeSO_4 \cdot 7H_2O$	0.001
$Na_2HPO_4 \cdot 12H_2O$	1.5
KH_2PO_4	1.5
pH	7.2

Table S3. Composition of PCR mix for amplification of the 16S rRNA encoding gene fragment.

Component	Volume [µL]	Concentration
5 × GoTaq Flexi Buffer (Promega, USA)	10	1 ×
$MgCl_2$, 25 mM (Promega, USA)	3	1.5 mM/µL
dNTP Mix, 25 mM (Blirt, Poland)	0.4	0.2 mM/µL
Primer 27f 10 µM (Sigma-Aldrich, Germany) (5'-AGA GTT TGA TCC TGG CTC AG-3')	2.5	0.5 µM/µL
Primer 1492r 10 µM (Sigma-Aldrich, Germany) (5'-TAC GGT TAC CTT GTT ACG ACT T-3')	2.5	0.5 µM/µL
GoTaq DNA Polymerase (5 U/µL) (Promega)	0,3	1.5 U/µL
DNA from bacteria	3	0.2 µg/µL
Nuclease-free water (Promega, USA)	28.3	-
Final volume	50	-

Table S4. Reaction conditions for amplification of the 16S rRNA encoding gene fragment.

Phase	Temperature [°C]	Time [min]	Number of cycles
Initial denaturation	95	5	1
Denaturation	95	1	30
Attachment	54	30	30
Elongation	72	2	30
Final elongation	72	5	1
Cooling	4	90	1

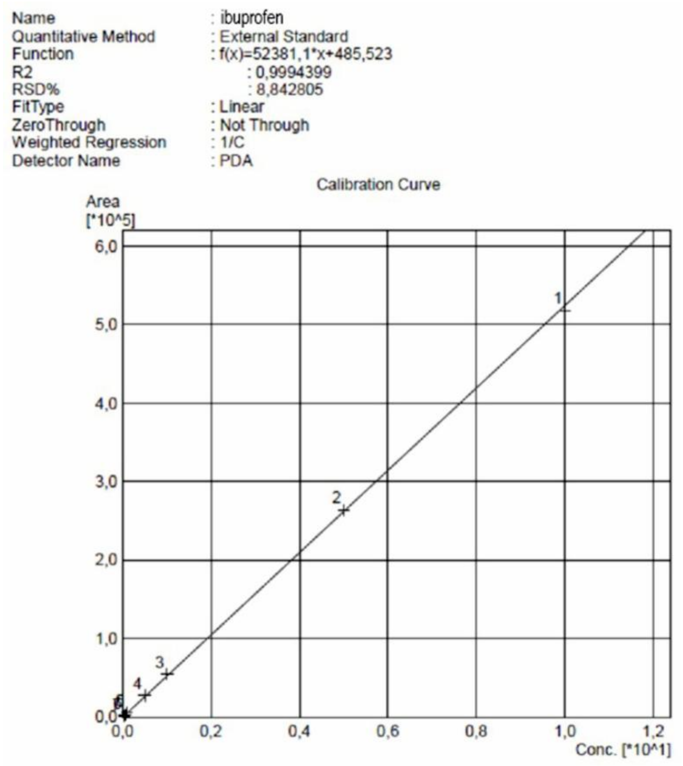

Figure S1. Calibration curve for working solutions of ibuprofen standard in the concentration range of 0.025–10 µg/mL, obtained during the validation procedure of the analytical method used to determine NSAID concentrations in MSM and soil.

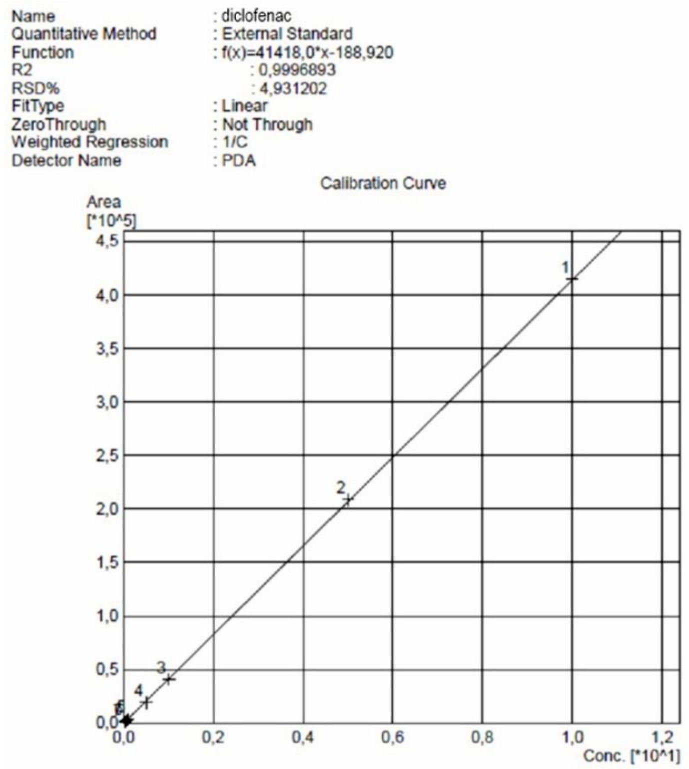

Figure S2. Calibration curve for working solutions of diclofenac standard in the concentration range of 0.025–10 µg/mL, obtained during the validation procedure of the analytical method used to determine NSAID concentrations in MSM and soil.

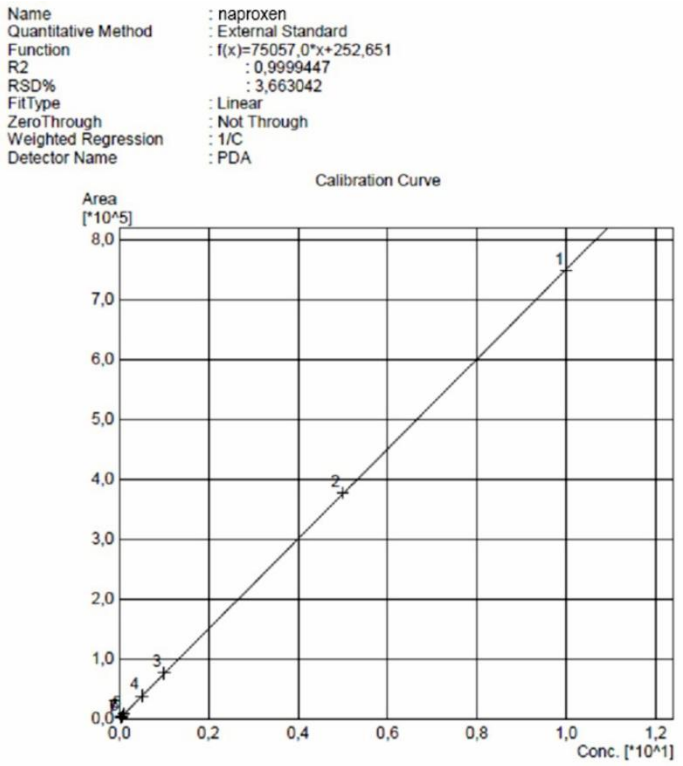

Figure S3. Calibration curve for working solutions of naproxen standard in the concentration range of 0.025–10 µg/mL, obtained during the validation procedure of the analytical method used to determine NSAID concentrations in MSM and soil.

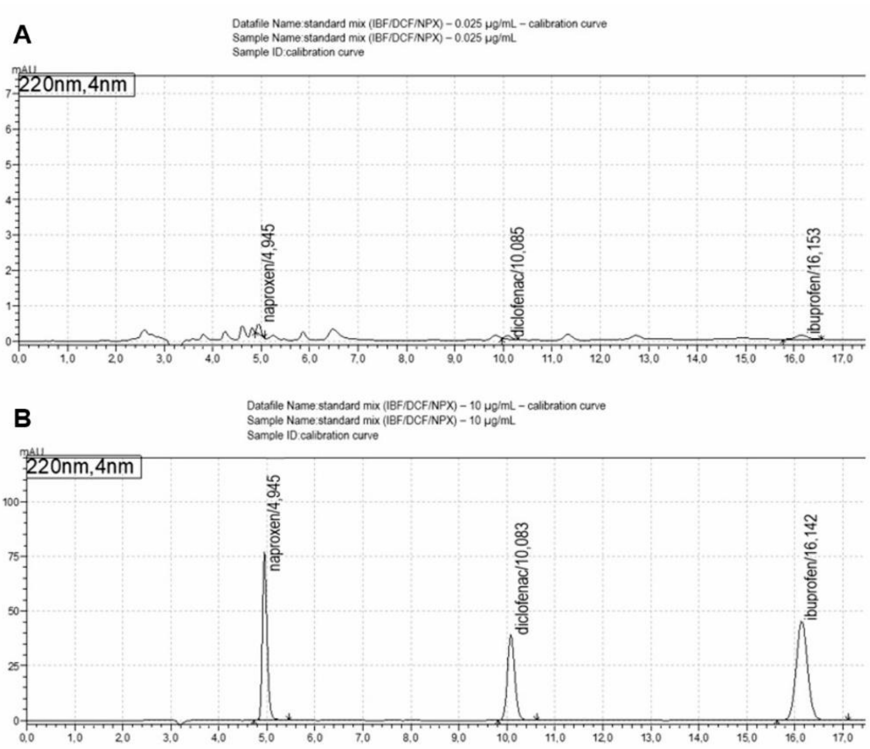

Figure S4. Example chromatograms for working solutions of ibuprofen, diclofenac and naproxen standards at concentrations of 0.025 µg/mL (A) and 10 µg/mL (B) used to prepare the calibration curve, and obtained during the validation procedure of the analytical method used to determine NSAID concentrations in MSM and soil.

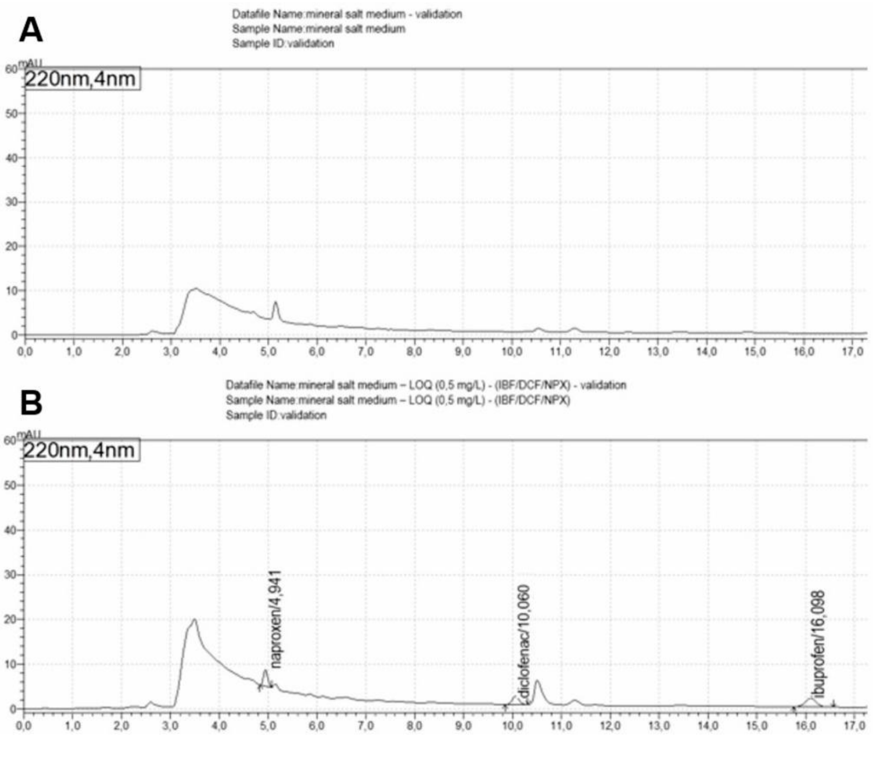

Figure S5. Example chromatograms for MSM samples – control (A) and with the addition of ibuprofen, diclofenac and naproxen standards at the LOQ (0.5 mg/L) level (B) obtained during the validation procedure of the analytical method used to determine NSAID concentrations in MSM.

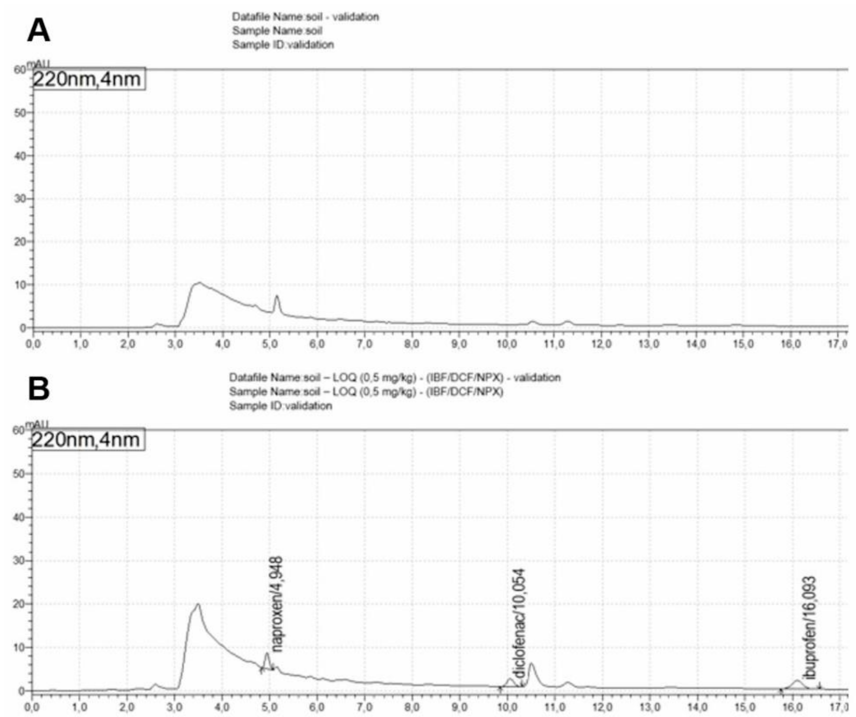

Figure S6. Example chromatograms for soil samples – control (A) and with the addition of ibuprofen, diclofenac, and naproxen standards at the LOQ (0.5 mg/kg) level (B) obtained during the validation procedure of the analytical method used to determine NSAID concentrations in soil.

```
LOCUS       OQ653250                1110 bp    DNA     linear   BCT 24-MAR-2
DEFINITION  Pseudomonas aeruginosa strain MC-1/23 16S ribosomal RNA gene,
            partial sequence.
ACCESSION   OQ653250
VERSION     OQ653250.1
KEYWORDS    .
SOURCE      Pseudomonas aeruginosa
  ORGANISM  Pseudomonas aeruginosa
            Bacteria; Pseudomonadota; Gammaproteobacteria; Pseudomonadales;
            Pseudomonadaceae; Pseudomonas.
REFERENCE   1  (bases 1 to 1110)
  AUTHORS   Cycon,M.
  TITLE     Direct Submission
  JOURNAL   Submitted (19-MAR-2023) Microbiology and Virology, Medical
            University of Silesia, Jagiellonska 4, Sosnowiec 41-200, Poland
COMMENT     ##Assembly-Data-START##
            Sequencing Technology :: Sanger dideoxy sequencing
            ##Assembly-Data-END##
FEATURES             Location/Qualifiers
     source          1..1110
                     /organism="Pseudomonas aeruginosa"
                     /mol_type="genomic DNA"
                     /strain="MC-1/23"
                     /db_xref="taxon:287"
                     /geo_loc_name="Poland: Katowice"
     rRNA            <1..>1110
                     /product="16S ribosomal RNA"
ORIGIN
        1 tacacatgca gtcgagcgga tgaagggagc ttgctcctgg attcagcggc ggacgggtga
       61 gtaatgccta ggaatctgcc tggtagtggg ggataacgtc cggaaacggg cgctaatacc
      121 gcatacgtcc tgagggagaa agtgggggat cttcggacct cacgctatca gatgagccta
      181 ggtcggatta gctagttggt ggggtaaagg cctaccaagg cgacgatccg taactggtct
      241 gagaggatga tcagtcacac tggaactgag acacggtcca gactcctacg ggaggcagca
      301 gtggggaata ttggacaatg ggcgaaagcc tgatccagcc atgccgcgtg tgtgaagaag
      361 gtcttcggat tgtaaagcac tttaagttgg gaggaagggc agtaagttaa taccttgctg
      421 ttttgacgtt accaacagaa taagcaccgg ctaacttcgt gccagcagcc gcggtaatac
      481 gaagggtgca agcgttaatc ggaattactg ggcgtaaagc gcgcgtaggt ggttcagcaa
      541 gttggatgtg aaatccccgg gctcaacctg ggaactgcat ccaaaactac tgagctagag
      601 tacggtagag ggtggtggaa tttcctgtgt agcggtgaaa tgcgtagata taggaaggaa
      661 caccagtggc gaaggcgacc acctggactg atactgacac tgaggtgcga aagcgtgggg
      721 agcaaacagg attagatacc ctggtagtcc acgccgtaaa cgatgtcgac tagccgttgg
      781 gatccttgag atcttagtgg cgcagctaac gcgataagtc gaccgcctgg ggagtacggc
      841 cgcaaggtta aaactcaaat gaattgacgg gggcccgcac aagcggtgga gcatgtggtt
      901 taattcgaag caacgcgaag aaccttacct ggccttgaca tgctgagaac tttccagaga
      961 tggattggtg ccttcgggaa ctcagacaca ggtgctgcat ggytgtcgtc agctcgtgtc
     1021 gtgagatgtt gggttaaktc ccgtaacgag cgcaaccctt gtccttagtt accagcacct
     1081 cgggtgggca ytctaaggar actgccggtg
```

Figure S7. The identification data of the MC-2/23 strain from GenBank.
